# Behavioral Studies of Zebrafish Reveal a New Perspective on the Reproductive Toxicity of Micro- and Nanoplastics

**DOI:** 10.3390/toxics12030178

**Published:** 2024-02-26

**Authors:** Baihui Wu, Haiyang Yu, Jia Yi, Pengyu Lei, Jiaxuan He, Jing Ruan, Peiye Xu, Runchao Tao, Libo Jin, Wei Wu, Qinsi Yang, Da Sun, Xiaoqun Zhang

**Affiliations:** 1 Institute of Life Science & Biomedical Collaborative Innovation Center of Zhejiang Province, Wenzhou University, Wenzhou 325035, China; 2 Zhejiang Provincial Key Laboratory for Water Environment and Marine Biological Resources Protection, School of Life and Environmental Science, Wenzhou University, Wenzhou 325035, China; 3 State and Local Joint Engineering Research Center for Ecological Treatment Technology of Urban Water Pollution, School of Life and Environmental Science, Wenzhou University, Wenzhou 325035, China; 4 Bioengineering College, Chongqing University, Chongqing 400030, China; 5 Wenzhou Institute, University of Chinese Academy of Sciences, Wenzhou 325000, China; 6 Key Lab of Biohealth Materials and Chemistry of Wenzhou, Wenzhou University, Wenzhou 325035, China; 7 Kaihua County Traditional Chinese Medicine Hospital, Quzhou 324399, China

**Keywords:** microplastics, nanoplastics, zebrafish, reproductive toxicity, neurobehavioral effects, environmental pollutants

## Abstract

The escalating prevalence of microplastics and nanoplastics in aquatic environments is a major challenge affecting the behavior and reproductive health of aquatic organisms while posing potential risks to human health and ecosystems. This review focuses on the neurobehavioral changes and reproductive toxicity of MNPs in zebrafish and their relationships. At the same time, the neurobehavioral changes caused by MNPs were studied, and the synergistic effects of the interaction of these pollutants with other environmental contaminants were explored. In addition, zebrafish, as a model organism, provide valuable insights into the subtle but important effects of MNPs on reproductive behavior, which is critical for understanding reproductive success, suggesting that behavioral changes can serve as an early biomarker of reproductive toxicity. In addition, based on classical endocrine disruptor models and behavioral research methods, the current status of the research on the reproductive toxicity of MNPs in zebrafish was reviewed, which further indicated that the behavioral parameters of zebrafish can be used as an effective and rapid tool to evaluate the reproductive toxicity of MNPs. However, behavioral methods for rapidly assessing the toxicity of MNPs are still an area of exploration. To address limitations and challenges in the current scope of research, this review outlines future research directions with the aim of improving our understanding of the environmental and health impacts of MNPs. This work aims to inform targeted environmental policies and advance public health strategies to address the growing challenge of MNPs pollution.

## 1. Introduction

The advent of plastic, a high-molecular-weight polymer, marked a revolutionary shift in modern society, attributed to its exceptional corrosion resistance, insulation properties, and ease of processing [1]. Since its inception in 1950, global plastic production has witnessed a meteoric rise, escalating from 3.8 million tons to a staggering 3.68 billion tons by 2019 [2]. This growth, while indicative of plastic’s utility, has precipitated severe environmental concerns. A 2019 study exemplifies this issue, revealing a concentration of 1794 pieces ·km^−2^ of plastic debris in the Antarctic Peninsula’s ocean surface, with microplastics (MPs) constituting over half of this contamination [3]. It is estimated that in the future, at least 5.25 trillion plastic particles, totaling more than 268,000 tons, will be discarded in the ocean based on data collected between 2007 and 2013 [4]. As these plastics enter the aquatic environment, they undergo photodegradation and weathering, fragmenting into smaller particles ranging from nanoscale to micron size [5]. This fragmentation results in the formation of MPs (100–5000 nanometers) and nanoplastics (NPs, 1–100 nanometers), which pose significant ecological threats [6,7]. The potential deposit, flotation, and adsorption of these micro- and nanoplastics (MNPs) in aquatic ecosystems are a significant issue [8] given that their small size enables easier absorption by organisms and the penetration of cell membranes, raising concerns for both marine life and human health [9,10].

In the marine environment, MNPs are exposed to sunlight, ultraviolet light, and visible light, leading to the production of reactive oxygen species (ROS). These ROS, including hydroxyl radicals (•OH), superoxide anion radicals (O_2_^•−^), and singlet oxygen (^1^O_2_), can cause the formation of oxygen-containing functional groups and polymer fractures on the surfaces of MPs [11,12]. This process enhances the ingestion of MNPs by microorganisms and other organisms. For example, polylactic acid (PLA) MNPs (irregular fragments; 5–50 µm), when exposed to ultraviolet light (90 days), have been shown to promote intake and accumulation in zebrafish [13]. Additionally, as MNPs degrade and weather, they may release more toxic compounds, such as additives and adsorptive organic pollutants, further exacerbating their impact [14,15].

The inherent characteristics of MNPs, such as their small particle size, high hydrophobicity, stability, and high mobility, make them capable of absorbing, aggregating, and carrying various pollutants [16]. They include persistent organic pollutants (e.g., polycyclic aromatic hydrocarbons, perfluorooctanoic acids, polychlorinated biphenyls, and polybrominated diphenyl ethers), heavy metals (e.g., arsenic, chromium, copper, cadmium, and lead), and pathogenic bacteria, which contribute to MNPs’ biotoxicity [17,18]. Numerous studies have consistently reported the biological toxicity of MNPs to aquatic organisms, encompassing developmental toxicity, neurotoxicity, reproductive toxicity, immunotoxicity, genotoxicity, and the potential for transgenerational transmission [19,20,21]. The reproductive system, a critical component for the survival and balance of aquatic ecosystems [22], is particularly susceptible to the adverse effects of MNPs [23].

The urgent need to develop efficient methods to assess the reproductive toxicity of MNPs in aquatic environments stems from the limitations of the current, time-consuming approaches. Recent studies have explored various methodologies, ranging from chronic exposure experiments in cladocerans to in vivo tests on testicular toxicity in mammals, highlighting the diverse impacts of MNPs on reproductive systems [24,25]. Investigations into the molecular mechanisms, such as the effects on oxidative stress and reproduction-related genes, provide deeper insights into the genetic-level impacts [26,27]. Furthermore, research on the transgenerational effects of MNPs, especially when combined with other pollutants, underscores the complexity of their ecological impact [28,29]. Studies have shown that NPs are able to penetrate cell membranes and cross the intestinal barrier, reach the bloodstream, and then travel to other organs to cause toxicity [30]. These studies collectively emphasize the need for rapid and comprehensive assessment methods to understand and mitigate the risks posed by MNPs.

Reproduction is crucial to the balance of an ecosystem. Invertebrates such as oysters [31] and nematodes [32] are being used as model organisms in many studies at the present time. Despite this, their reproductive systems are simpler than those of vertebrates, and their biological differences differ considerably from those of humans. Therefore, the results of the research may not be representative. As a result of the high cost of mice [33], their long reproductive cycle, and the always complex ethical issues that surround their use, these animals are not the best choice for experiments. However, as a typical representative of aquatic organisms, fish play an important role in a number of ecosystems and are crucial to maintaining food chains and ecological balance. Additionally, fish serves as the principal protein source for humans, and MNPs may be able to transmit pathogenic bacteria and viruses through the food chain [34,35,36]. Zebrafish, in particular, serve as an ideal model organism for environmental toxicology studies [37]. Their genetic similarity to humans and transparent embryos allow for direct observation and manipulation, making them suitable for assessing the impact of environmental pollutants [38,39]. Zebrafish exhibit a wide array of behavioral responses, such as social interaction, exploration, foraging, and reproduction, which are integral to their response to environmental changes [40]. These behaviors provide a rapid biomarker of the effects of environmental pollutants on the molecular, biochemical, and physiological systems of animals [41].

Studies have indicated that MNPs not only affect the reproduction of mammals but also induce changes in their reproductive behavior [31]. Normal social behaviors are essential for reproductive success, and maintaining stable and normal social interactions between males and females is crucial for proper reproduction (such as the amount of time and number of contacts between male and female fish) [42,43,44]. The sociological behavior of zebrafish mirrors that of humans, making behavioral studies relevant to assess the effects of chemicals on reproductive behavior and fertility in both animals and humans [45]. The current reproductive toxicity studies of MNPs in zebrafish follow the classical endocrine disruptor model, characterized by changes in fecundity, oviposition success, and hypothalamic–pituitary–gonadal (HPG) axis responses [46,47].

**Figure 1:**
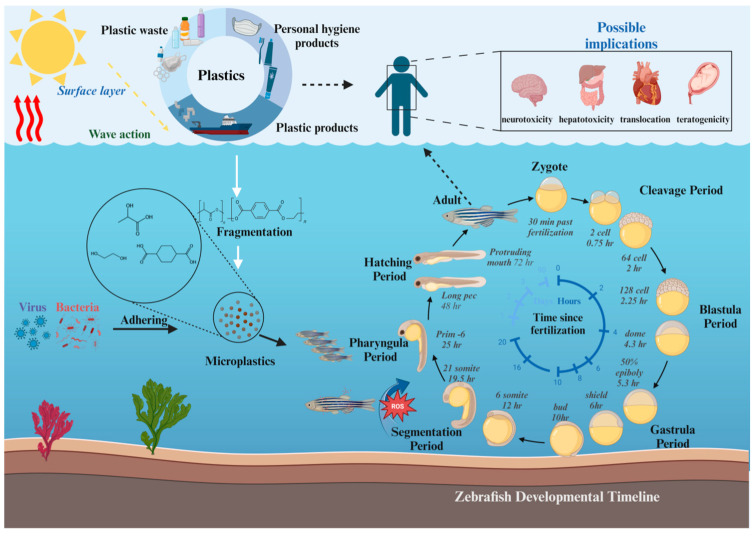
The environmental cycle of MNPs, including the source, decomposition, intake, and the impact of MNPs on humans and aquatic ecosystems.

This review aims to comprehensively examine the effects of MNPs on the behavior and reproductive system of zebrafish, with a focus on behavioral changes as a biomarker of reproductive toxicity and the interaction between behavioral changes and reproductive endocrine function. This review conducted a bibliographic survey of the reference papers by using various bibliographic databases such as PubMed, Google Scholar, and Web of Science. “Zebrafish” and “Reproductive toxicity” were used as keywords for retrieval, and the time span was nearly 10 years. About 300 articles were retrieved. “Zebrafish”, “Behavior”, and “Microplastics” were used as keywords for retrieval, and the time span was nearly 8 years. About 150 articles were retrieved, and after careful consideration, 167 articles were finally selected for inclusion in this review. The selection process was based on relevance to the topic, credibility of the source, and recentness of the publication. Papers that did not meet these criteria were excluded to ensure the quality and relevance of the review. We endeavor to elucidate the specific effects of MNPs on zebrafish reproduction and explore their synergistic effects with other environmental pollutants. Additionally, we present the current research methods and technological progress in this field, providing a scientific basis for environmental policy making and public health protection. Our aspiration is that this review will deepen our understanding of the environmental and health impacts of MNPs and foster further scientific research and discussion in related fields. And, Figure 1 shows the environmental cycle of MNPs, including the source, decomposition, intake, and the impact of MNPs on humans and aquatic ecosystems.

## 2. Association of Behavioral Changes with Reproductive Toxicity

### 2.1. Behavioral Changes as Early Biomarker of Reproductive Toxicity

Reproductive toxicity in marine animals encompasses a spectrum of adverse effects on various stages of the reproductive cycle, including gametogenesis, gamete and oocyte quality, fecundity, egg production, and sperm motility [48]. Zebrafish, a sensitive biomarker species, exhibit behavioral responses to environmental stressors such as exposure to MNPs, which can manifest as significant alterations in swimming, social interaction, and courtship behaviors. These behavioral changes are often precursors to impaired fertility [49].

Zebrafish exhibit a range of intricate courtship behaviors, such as males rapidly approaching females, nudging them with their mouths, leading them to spawning sites, and displaying complex swimming patterns like circling or figure-eight movements. These behaviors, crucial for successful reproduction, are accompanied by body oscillations at varying frequencies and amplitudes, facilitating the release of sperm and egg [50]. Male zebrafish also exhibit territorial behavior during mating, staying within close proximity to the spawning site and warding off other males, underscoring the importance of these behaviors in the reproductive process [51]. A study by Lan et al. found that male zebrafish exposed to tributyltin showed a reduced gathering frequency, frequency of visits, and time spent in spawning areas, which affected reproduction [44]. Similarly, Hou et al. discovered that after 60 days of exposure to 398.6 ng/L of the Norethindrone (NET), male zebrafish spent less time participating, tracking, and mating. The effects of NET on females can result in virilization, causing males to show less intimacy and mating interest toward females exposed to NET [52]. Yan et al. reported that tebuconazole inhibits the spawning volume and fertilization rate of zebrafish by interfering with their social behavior [53].

MNPs, known to impact the nervous system, can cause behavioral changes in zebrafish [54]. Exposure to MNPs has been associated with alterations in swimming behavior and anxiety-like behaviors in adult fish [55]. For instance, NPs and bisphenol AF exposure significantly reduced the average velocity and total distance traveled by parents as well as the number of eggs laid by female zebrafish [56]. High concentrations of polystyrene NPs (PS-NPs) led to notable changes in locomotor activity, aggression, shoaling behaviors, and predator avoidance [49].

The reproductive process in zebrafish involves a series of behavioral steps, including courtship, chase, breeding, egg release, and sperm release. MNPs have the potential to adversely affect any of these stages [57]. Therefore, observing behavioral changes in zebrafish after exposure to MNPs provides a rapid and efficient method for the early detection of potential reproductive toxins, enabling appropriate measures to ensure reproductive health and contributing to a better understanding of the environmental fate and ecological impact of MNPs in aquatic environments.

### 2.2. Behavioral and Reproductive Endocrine Interactions

MNPs, acting as endocrine disruptors, not only influence the reproductive behavior of aquatic organisms but also significantly impact hormone levels, which are crucial for the development and function of reproductive organs [58,59,60]. In zebrafish, a model organism for studying these effects, the disruption of endocrine balance plays a pivotal role in behavioral changes [61]. During mating, male zebrafish engage in behaviors like chasing females and using specific swimming movements to attract their attention, which are essential for successful fertilization. Environmental pollutants, including MNPs, can influence these behaviors, which are also affected by factors like temperature and light.

Upon acceptance of the males’ courtship, zebrafish engage in copulation, leading to the release of sperm and eggs and subsequent fertilization [42,62,63]. This process is not unique to aquatic animals; for example, the male golden-collared parrot (*Manacus vitellinus*) performs acrobatics during courtship to attract mates and increase reproductive success [64]. The social behavior of fish, including zebrafish, is integral to their reproductive success. Exposure to MNPs can adversely affect critical aspects of social behavior, such as the timing and number of contacts between males and females, which are vital for mate choice and reproduction [65]. Stress and anxiety behaviors in zebrafish, potentially induced by MNPs and other pollutants like copper, may lead to reproductive endocrine disorders, altering behaviors that are crucial for survival, foraging, and reproduction [66].

The gonadal glands produce hormones such as estrogen, androgens, and progesterone, which govern behavior in reproductive and social contexts in vertebrates [67]. MNPs have been found to affect zebrafish sex hormones. For instance, combined exposure to triphenyl phosphate (TPhP) and MNPs can alter steroid hormone and vitellogenin (VTG) levels, significantly affecting the egg count, fertilization rate, and hatching rate [68]. Male sexual behavior, heavily dependent on hormones like testosterone (T), is disrupted by a lack of T, affecting both desire and behavioral phases [69]. Similarly, estrogen and progestin, secreted by the ovaries, promote sexual behavior in female animals during estrus [67].

Territorial acquisition by males is also crucial for mating. MNPs can mimic natural hormones, reducing circulating androgen levels in male fish and thereby diminishing aggression and competitive behavior, which are essential for successful spawning [70]. A decrease in courtship behavior or reduced pairing success can indicate an impaired reproductive capacity in zebrafish. Therefore, studying the effects of reproductive toxic substances requires a comprehensive consideration of their impacts on the reproductive system, nervous system, and any behavioral changes that may result [53].

**Figure 2:**
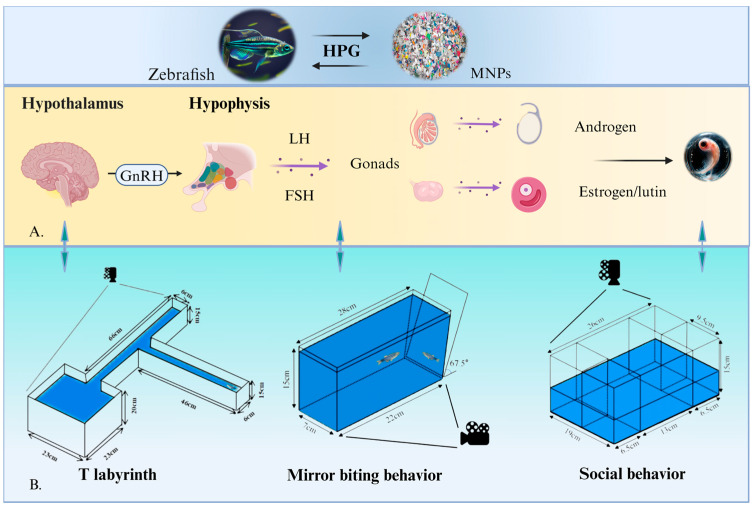
Behavioral changes interact with reproductive and endocrine systems in zebrafish. (**A**) MNPs affect endocrine level changes; (**B**) experimental setup for behavioral studies of common zebrafish.

The interaction between the behavior and reproductive endocrine system in zebrafish highlights the potential threat posed by MNPs to reproductive health (Figure 2). These findings provide a crucial scientific basis for future research and environmental protection policies regarding the reproductive toxicity of MNPs.

## 3. Effects of MNPs on the Behavior of Zebrafish

### 3.1. Short-Term and Long-Term Effects of MNPs’ Exposure on Fish Behavior

MNPs, due to their adverse effects on the nervous system, can lead to behavioral abnormalities in animals. For example, Sarasamma et al. demonstrated that a 7-day exposure of zebrafish to 1.5 mg/L PS-NPs (~70 nm) resulted in increased exploratory behavior and attenuated aggressive and predator-avoidance behaviors, which are crucial for reproductive success [49]. This exposure also caused disturbances in lipid and energy metabolism, oxidative stress, and tissue accumulation in zebrafish. Perersen et al. found that zebrafish embryos exposed to 100 and 1000 μg/L PS-NP (200 nm) over five days exhibited significant changes in genes related to nervous system function and motor behavior, suggesting a primary toxic effect on neurobehavioral functions [71].

Various experiments, such as light–dark stimulation experiments, tapping experiments, open field experiments, and new tank experiments, are employed to study the neuromotor behavior of zebrafish [72]. Interestingly, zebrafish possess spatial memory and learning abilities and are highly sensitive to stressful stimuli, which manifest in behaviors like anxiety, mate choice, aggression, and social behavior [73]. Zhu et al. observed a dose-dependent decrease in female fecundity in Japanese rice fish (*Oryzias latipes*) after long-term exposure to 500, 1000, and 2000 μg/g fluorescent spherical PS-MPs (10 μm) for 10 weeks [74]. Adult zebrafish exposed to 10 and 100 μg/L (0.10–0.12 μm) PS-MPs for 35 days showed pathological changes in brain tissue, including inflammatory cell infiltration, degeneration, cytoplasmic vacuolization, and neuronal death [75].

Swimming ability, vital for predation, mating, and defense throughout a fish’s life, is also impacted by MNPs. Exposure to 100 and 1000 μg/L PS-NPs (1 μm, around 1.91 × 10^6^ and 1.91 × 10^7^ particles/L) resulted in a 4.6% and 2.6% decrease in swimming distance and a 4.9% and 2.8% decrease in activity speed, respectively [76]. African catfish (*Clarias gariepinus*) exposed to 0.5, 1.0, and 1.5 g of low-density polyethylene (PE) showed an affected survival and swimming ability [55]. Short-term exposure (4–96 hpf) to MPs (1 or 10 mg/L) in the early life stage of zebrafish resulted in only weak toxic effects, whereas long-term (4 months) exposure led to a reduction in the body weight of the parents and their offspring’s swimming behavior [77]. Therefore, MNPs do not only affect the parents but also affect the growth and behavior of their offspring through their accumulation [46,78].

In zebrafish, acute exposure to MNPs may result in increased stress behavior or decreased mobility while prolonged exposure can lead to more complex behavioral changes, such as a decline in learning and memory abilities, indirectly affecting reproductive behavior and success. Thus, both short-term and long-term exposure to MNPs affect fish behavior in various ways, impacting the nervous system, metabolism, social behavior, and ultimately reproductive capacity.

### 3.2. Exploration of the Physiological Mechanisms of Behavioral Change

The primary abnormalities exhibited by animals after MNP exposure are neurobehavioral in nature. Current studies have proposed several mechanisms responsible for MNP toxicity, including oxidative stress [79], immune responses [80], and changes in energy metabolism [81]. Given the variety of observed behavioral responses, it is likely that other processes are also involved [82]. MNPs pose a significant threat to the endocrine system due to their properties as endocrine disruptors. They can interfere with the normal functioning of the endocrine system by impeding the binding of hormones to their receptors, thereby affecting hormone signaling in the body [83]. This can result in abnormalities in intracellular signaling pathways that ultimately affect behavior. For example, PS-MPs have been shown to bind to androgen receptors (ARs), estrogen receptor α (ERα), and estrogen receptor β (ERβ) with binding energies comparable to those of natural ligands like T and estradiol (E_2_). However, these MNPs have more amino acids and hydrogen bonds, leading to a stronger binding affinity with receptors and potential endocrine disruption [84,85].

Oxidative stress is a common mechanism of action for endocrine-disrupting chemicals (EDCs)-induced toxicity. MNPs can cause oxidative damage to specific regions of the brain, posing developmental risks and negatively affecting neurological development [60,86]. An imbalance between the production of ROS and the ability of antioxidant enzymes to remove them can occur as a result of oxidative stress. Studies have examined the effects of MNP exposure on gene expression and on the activity of antioxidant enzymes such as catalase (CAT), superoxide dismutase (SOD), glutathione peroxidase (GPX), peroxidase, and glutathione reductase [87]. For instance, exposure to 10–100 μg/L PS-MPs could significantly increase ROS levels and affect the expression of CAT, SOD1, and GPX1a related to oxidative stress in zebrafish [88]. There was a significant increase in the activities of SOD and CAT due to PS-MPs exposure [89]. Moreover, they play a crucial role in mitigating the negative effects associated with excessive ROS production [90]. ROS accumulation can interfere with the functioning of the nervous system in various ways. During oxidative stress, neurons may experience oxidative damage and apoptosis, accelerating the degenerative process and triggering neuroinflammation, which affects the normal conduction and function of neurons [91]. Behavioral disorders are also influenced by the occurrence of oxidative stress in vivo. MNPs with a diameter of 20 nm can enter and accumulate in the zebrafish brain, producing excessive ROS and resulting in behavioral disorders and brain damage [92]. Zebrafish were significantly affected by oxidative stress in terms of locomotor activity, aggression, shoal formation, and predator-avoidance behaviors [49].

Furthermore, MNPs can affect the normal function of the nervous system by impacting the synthesis, release, and reuptake of neurotransmitters, subsequently affecting behavior. Exposure to MNPs can alter gene expression related to neurodevelopment and transmission. MNPs in the brain can inhibit acetylcholinesterase (AChE) and change the neurotransmitters’ levels and the content of neurotransmitters such as dopamine and acetylcholine, leading to neurotoxicity [93]. For example, exposure to PS-MPs affected the levels of dopamine and acetylcholine in goldfish, as well as the expression of the genes related to neurotransmission [94]. The movement distance of zebrafish larvae was significantly reduced in response to exogenous dopamine (100 μm), indicating that abnormal dopamine content interferes with animal behavior [95]. The inhibition of *Nile tilapia* AChE activity was observed after exposure to PS-MPs (1–100 μg/L), with a maximum inhibition rate of 37.7% [96]. Similar effects were observed in zebrafish, where exposure to PS-MPs affected motor behavior, AChE activity, and gene expression related to AChE [79]. Zebrafish embryos exposed to 50 mg/L of 1 nm PS-NP developed central nervous system dysfunction and abnormal motor activity [79]. PS-NPs of 5 nm at a concentration of 1 mg/L altered the levels of 11 neurotransmitters in adult zebrafish, affecting their predator-avoidance and aggression behavior [49].

Researchers have hypothesized that behavioral changes in fish (*Symphysodon aequifasciatus*) following exposure to MNPs (88 nm and 900 μm) are attributed to changes in brain–gut–microbiota interactions [97]. Although the focus of research varies, there is a consensus that MNPs can cause neurotransmitter system disorders and result in abnormal behavior.

## 4. Effects of MNPs on the Reproductive System of Zebrafish

### 4.1. Direct Effects of MNPs on Reproductive Organs and Reproductive Endocrine Effects

**Figure 3:**
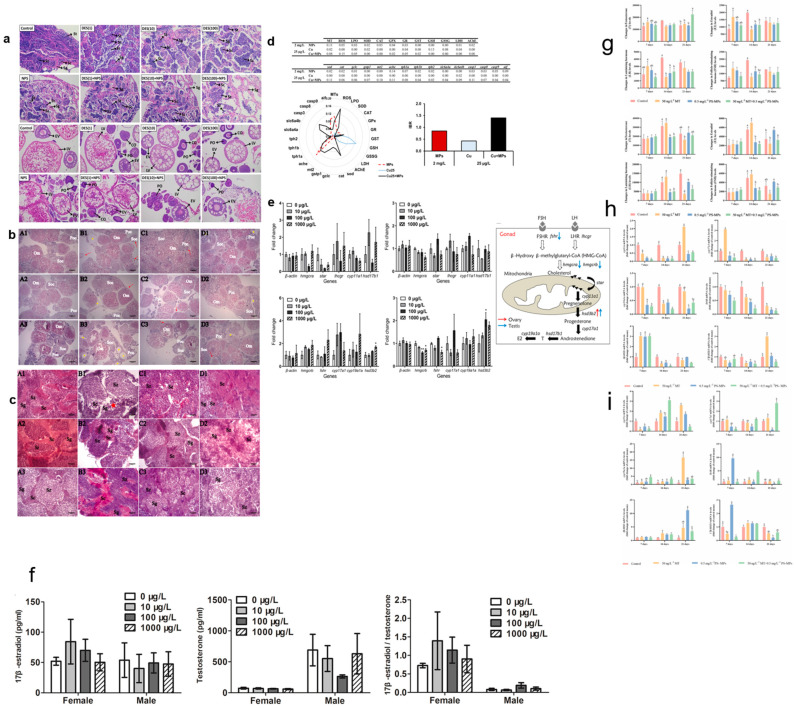
Reproductive effects of MNPs or MNPs and other pollutants on zebrafish. (**a**–**c**) Morphological changes in reproductive organs [100,101]; (**a**) copyright © 2022 Elsevier; (**b**,**c**) copyright © 2023 published by Elsevier; (**f**–**h**) changes in hormone levels [101,102]; (**f**) copyright © 2020 Elsevier; (**g**,**h**) copyright © 2023 Elsevier; (**d**,**e**,**i**) reproductive hormone gene expression [101,102,103]; (**d**) copyright © 2022 Elsevier; (**e**) copyright © 2020 Elsevier; (**i**) copyright © 2023 Elsevier. Spermatogenic cycle include spermatogonia (Sg), spermatocytes (Sc), spermatids (St), and spermatozoa (Sz). perinuclear oocytes (PO), cortical alveolar oocytes (CO), early vitellogenin oocytes (EV) and late vitellogenin oocytes (LV). Om is the ovum, Soc is the secondary oocyte, and Poc is the primary oocyte. The pentagram represents the vacuolated oocyte, and the red arrow represents the oocyte’s missing linkage with the follicular cell layer. * *p* < 0.05, ** *p* < 0.01; the different letters represent the significance level (*p* < 0.05).

MNPs impact reproduction not only through behavioral pathways but also directly through the reproductive system. They can translocate from the oral–gastrointestinal tract or be absorbed through gills and transdermal pathways, accumulating in organs such as gills, intestines, and gonads via the circulatory system [98]. Once inside germ cells, tissues, and organs, MNPs can alter the normal morphology, histology, and physiological function of the reproductive system [31,46]. Sarasamma et al. found that gonads were among the major tissues enriched in MPs after zebrafish exposure [49]. Exposure to PS-MPs for 60 days resulted in histological changes in the ovary, cell apoptosis via the Sirt1-p53 pathway, and altered levels of plasma steroid hormones (E_2_/T ratio) [84]. MNPs induce molecular responses and histological changes in fish gonads. In zebrafish, changes such as the reduced testicular basement membrane thickness in males, altered expression of apoptosis-related genes (*p53*; *Bax*; *Caspase-7*, *-8*, and *-9*), and increased levels of spermatocyte apoptosis have been linked to spermatogenesis disorders [46]. The reproductive systems of other fish species are also adversely affected by MNPs. Wang et al. found that the exposure of marine *Oryzias melastigma* to PS-MPs (2, 20, and 200 μg/L) for 60 days delayed gonad maturation in female fish and delayed hatching in parental fish exposed to 20 μg/L. There was also a downregulation of the genes involved in the female steroidogenic pathway, leading to decreased concentrations of E_2_ and T levels in female plasma [99]. In mature female *Oryzias latipes* exposed for 10 weeks to PS-MPs, egg production was reduced [74]. Figure 3 shows the effects of MNPs or MNPs and other pollutants on zebrafish reproduction (histological changes in reproductive organs, hormone levels, and reproductive hormone gene expression).

MNPs can adversely affect the reproductive system in various ways, including via interference with hormones, inflammatory responses, cell damage, and reproductive toxicity. Factors influencing the toxicity of MNPs include temperature, light, humidity, and chemicals. Increasing the temperature and the amount of light exposed to MNPs will accelerate the decomposition process of MNPs and can have an adverse effect on the reproduction of organisms [13]. The photodegradation of PLA in laboratory simulations elevated reproductive toxicity, including the abnormal differentiation of oocytes, altered levels of sex hormones, and altered ovarian tissue metabolism [104,105]. Plastic additives, including plasticizers, antioxidants, UV stabilizers, dyes, and flame retardants, which account for a significant portion of plastics, can negatively impact organism reproduction. Approximately 1000 of these additives are EDCs [106], which mimic or interfere with endogenous hormones, affecting the normal function and development of the reproductive system [85,107,108,109].

EDCs have been shown to negatively impact zebrafish reproduction, including dysregulated sex ratios, immature gonads, reduced sexual behavior, decreased sperm count, and lowered oviposition and fertilization rates. These are mostly caused by the disruption of sex steroid hormones by endocrine disruptors [110]. Some MNPs contain chemicals that cause estrogenic effects and the abnormal development of the reproductive system in male animals, leading to decreased fertility. The excessive bioaccumulation of MNPs can lead to increased spermatogenesis apoptosis and disrupted spermatogenesis [111]. Furthermore, MNPs may result in a hormonal imbalance in organisms, leading to abnormal levels of hormones and interference with the normal function of the endocrine system. This may have a negative impact on the development and function of the reproductive system. Significant changes in androgen and estrogen metabolism and biosynthesis have been observed in organisms exposed to PE-MPs, suggesting an effect on the regulation of key hormones such as estrogen, androgen, and thyroxine [112].

Vitelogenin plays a vital role in zebrafish reproduction [113]. MNPs can impair the expression of the phase 1 detoxification-related gene (cytochrome P450 1A, *cyp1a*) and the oogenesis-related gene vitellogenin 1 (*VTG1*), affecting VTG supply and impaired oogenesis [114]. The VTG can promote the transfer of MNPs from the oocyte to the yolk sac of the embryo, thereby passing the plastics to the offspring [78]. MNPs significantly upregulated the expression of *VTG1* in the liver of male zebrafish, feminizing them [115]. Besides, MNPs’ accumulation in the ovaries and granulosa cells can lower the levels of anti-Mullerian hormone and E_2_, leading to an irregular estrous cycle and abnormal folliculogenesis [116,117].

Researchers believe that the reproductive toxicity of MNPs is caused by three factors: damage to the intestinal tract mucosa, which disturbs the intestinal flora and affects food digestion and absorption [118,119,120]; gonad absorption to compensate for the decreased energy balance, leading to the gonads becoming energy reserves [49]; and accumulation in gonadal tissues, interfering with the expression of genes related to the endocrine system or gonads. This can reduce the levels of hormones like luteinizing hormone (LH), follicle-stimulating hormone (FSH), and T; destroy the blood–testis barrier; and lead to testicular inflammation, atrophy, and degeneration, as well as abnormalities in sperm quality [99,121,122]. MNPs also significantly reduce the expression of the genes involved in vitellogenesis, especially *VTG1* and *VTG2* [121]. They may produce oxidative stress through ROS, triggering biological responses detrimental to reproductive health, including inflammation, apoptosis, and signaling pathways induced by oxidative stress. MNPs can cause oxidative stress in zebrafish ovarian cells, increase DNA damage, and induce an increase in apoptosis in genes associated with vitellogenesis [47,121]. Additionally, MNPs significantly upregulate proinflammatory genes (NFκβ and TNF-α), affecting the normal ovulation process. By affecting the Sirt1-p53 pathway, which plays an important role in cell stress response and apoptosis, MNPs disrupt related signaling pathways and negatively affect reproduction [121].

**Figure 4:**
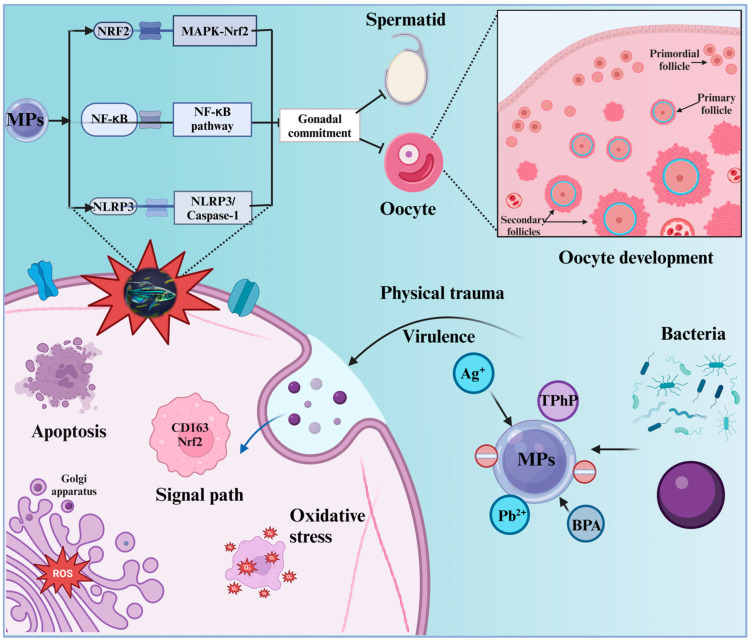
Mechanism of reproductive toxicity induced by MNPs in zebrafish and related signaling pathways (MAPK-Nrf2, Nrf2/HO-1/NF-κB, p38 MAPK, and NLRP3/Caspase-1).

MNPs also cause reproductive toxicity through mechanisms other than oxidative stress [123] and are involved in many signaling pathways, including those involved in apoptosis [46]; endoplasmic reticulum stress [124]; and the MAPK-Nrf2 [125], Nrf2/HO-1/NF-κB [117], p38 MAPK [126], and NLRP3/Caspase-1 signaling pathways (Figure 4) [127]. Sex hormone levels in adult zebrafish are altered by modifying the expression of regulatory genes in the HPG axis, ultimately affecting reproductive performance. Gender is a factor affecting the interference effect [128]. Through the regulation of SIRT1, PS-MPs (0.5 µm) impair female reproduction in zebrafish by causing oxidative stress, apoptosis, and hormonal imbalance. Exposure to PS-MPs reduces the reproductive rate in female zebrafish, increases apoptotic signaling and ROS levels, and causes morphological changes in the gonads. These changes also affect the ratio of E_2_/T in plasma [84]. Through the SIRT1/p53 pathway, PS-MPs adversely affect fertility, gonadal morphology, steroidogenesis, and the HPG axis. The HPG axis, an important endocrine regulatory pathway for fish reproduction, controls the production of sex hormones such as E_2_ and T [129]. MNP exposure negatively influences the HPG axis in women, downregulating genes pertaining to the steroidogenic pathway and resulting in a reduction in E_2_ and T levels in female plasma, leading to endocrine system disorders [99]. MNPs may interfere with the reproductive process of fish by altering the secretion of sex hormones and the relative expression of genes related to the HPG axis. However, the molecular mechanisms underlying sex-specific MNPS-induced reproductive toxicity in zebrafish have not been fully explored and warrant further investigation.

### 4.2. Synergistic Effects of MNPs with Other Pollutants

The interaction between MNPs and other environmental pollutants can lead to synergistic effects, amplifying their impact when combined. MNPs, due to their small particle size and strong hydrophobicity, are adept at adsorbing other pollutants, including persistent and accumulative toxic substances like metal ions, polycyclic aromatic hydrocarbons, polychlorinated biphenyls, and antibiotics [130]. These pollutants can adhere to the surfaces of MNPs, increasing the risk of harm to aquatic organisms and potentially impacting human health through the food chain [131]. The aging of plastics can create more uneven surfaces, enhancing their adsorption capacity. For example, under UV lamp irradiation, aged PE MPs, PS-MPs, and polyamide microplastics (PA-MPs) showed increased adsorption to Cr, potentially enhancing their overall adsorption capacity [132].

The combined effects of MNPs and heavy metals in organisms can be synergistic or additive, enhancing bioaccumulation and toxicity [133]. For instance, MPs have been found to increase the degree of oxidative damage and inflammation induced by Cd in zebrafish tissues, affecting social and courtship behavior and ultimately impacting reproductive success [20,121,134].

As listed in Table 1, the interaction of MNPs with persistent organic pollutants can alter levels of VTG and sex hormones in fish [135]. When PS-MPs adsorb EDCs, they are more likely to penetrate the fish [136], accumulating in gonadal tissue and inhibiting the normal development of egg cells, spermatogonia, and spermatocytes [137]. This can lead to the abnormal morphology and function of gonadal tissue, exacerbating pathological damage to the gonads and impacting fish reproductive behavior [138]. It has been demonstrated that NPs can promote TPhP-induced gonadomegaly in zebrafish. By adding MPs and NPs to TPhP alone, further inhibition of the process of spermatogenesis and oogenesis was observed, which even resulted in significant reductions in the number of oocytes in the atretic follicles of the testis and ovary. The combination of TPhP with PS-MPs significantly reduced the number of eggs laid, fertilization rates, and hatching rates [68]. Consequently, combined exposure to MNPs and pollutants may increase fish reproductive toxicity.

In conclusion, the reproductive toxicity of MNPs in zebrafish reveals that the synergistic effects of MNPs with other pollutants significantly exacerbate their impact on the reproductive system and behavior of these aquatic organisms. This finding is crucial in understanding the broader implications of MNP pollution in aquatic environments. It highlights the urgent need for targeted environmental strategies that address not only the reduction in MNP pollution but also the complex interactions between these pollutants and other environmental contaminants. Such comprehensive approaches are vital for protecting aquatic life and maintaining the ecological balance of our water ecosystems. The detailed effects of MNPs and their combination exposure with other contaminants on the reproductive system of zebrafish are summarized in Table 1.

## 5. Research Methods and Technical Progress

In the realm of studying the effects of MNPs on zebrafish, research methods and technical advancements have significantly evolved, offering a nuanced understanding of the complex interactions between environmental pollutants and biological systems. Behavior analysis in zebrafish, encompassing responses to internal and external stimuli, serves as a critical biomarker of the molecular, biochemical, and physiological effects of these pollutants [72,142].

Current methodologies in zebrafish behavioral studies are diverse, integrating both movement and spatial biomarkers to capture a comprehensive picture of life activity. The movement index, such as the distance traveled, speed, and angular velocity, describes the physical capabilities and activity levels of zebrafish [143]. Spatial biomarkers, on the other hand, focus on the time spent in different areas, entries, and exits and are indicative of spatial distribution, preference, and emotional states like panic and anxiety [144].

Extensive research on zebrafish behavior has delved into various aspects, including motor behavior (e.g., swimming distance, average speed, and swimming speed), social interactions, memory and learning, anxiety-related behavior, and courtship behavior. Courtship behaviors in zebrafish, critical for reproduction, include approaches, escorting, displaying, leading, and laying eggs for females and chasing, tail-sniffing, zigzagging, and trembling for males [145]. A growing body of research quantifying zebrafish courtship behavior (chasing, zigzag, tail scent, and trembling) has demonstrated that male zebrafish lacking androgen receptors exhibit significantly reduced courtship behavior, thus demonstrating the important role played by this receptor in driving courtship [146]. As an example, MNP exposure may reduce the intracellular translocation of androgen receptors (ARs) mediated by testosterone, thereby decreasing sexual development and desire in males [147]. Moreover, zebrafish courtship behavior is also associated with appetite, oxygen content, and density. It has been shown that MNP exposure can significantly reduce appetite-associated endocannabinoid receptor type I (cnrl) and anandamide levels. MNP exposure can also reduce fecundity and courtship behavior due to Gnrh2 depletion [148,149]. MNPs also altered the expression of genes encoding G protein-coupled receptors in the olfactory bulb, resulting in olfactory bulb damage, and males with anosmia were incapable of wooing females [50,94]. Furthermore, the synergistic toxicity of MNPs and triclosan resulted in a decrease in oxygen uptake, and low oxygen levels influenced male zebrafish aggression, a behavior closely related to territorial occupation during breeding [150,151].

Social behavior is another important factor in determining the success of zebrafish reproduction. Photoperiodic effects on zebrafish spawning behavior are evident as they exhibit unique diurnal activity patterns that are synchronized with lighting patterns and feeding patterns [152]. In zebrafish, both PGA and PLA may alter circadian rhythm behavior and induce anxiety by affecting the brain-derived neurotrophic factor [153]. Female zebrafish exhibiting anxiety behavior exhibited more shy behavior, such as decreased access to the central area and more episodes of freezing. Male zebrafish that exhibit anxiety-like traits show motor decline and decreased curiosity. However, boldness is associated with a variety of behaviors, including increased activity, exploration, aggression, dispersal, and even the choice of a mate [154]. A decrease in social preference could be attributed to other factors, such as MNPs’ effects on sensory organs and the central nervous system, resulting in a hormonal imbalance and, ultimately, reproductive toxicity [61].

Factors affecting zebrafish behavior include external conditions and the zebrafish themselves. For this reason, when conducting behavioral studies of zebrafish, these factors must be taken into account and controlled to ensure that the results are reliable and accurate.

Developing zebrafish are a preferred vertebrate for high-throughput screening (HTS) due to their cost-effectiveness and transparency [155]. Over the past few years, screening forms targeting a growing number of pathways and endpoints, including endocrine disorders, have been designed and developed [156]. Zebrafish screening can be used to test a wide range of environmental poisons, pharmaceutical agents, and chemical libraries, covering a range of life stages, transgenic and mutant strains, concentrations, and exposure times [157]. In this study, zebrafish will be exposed to different concentrations and exposure times in order to study their behavior. Furthermore, animal behavior research involves experimental video recording and a quantitative analysis of video references.

**Figure 5:**
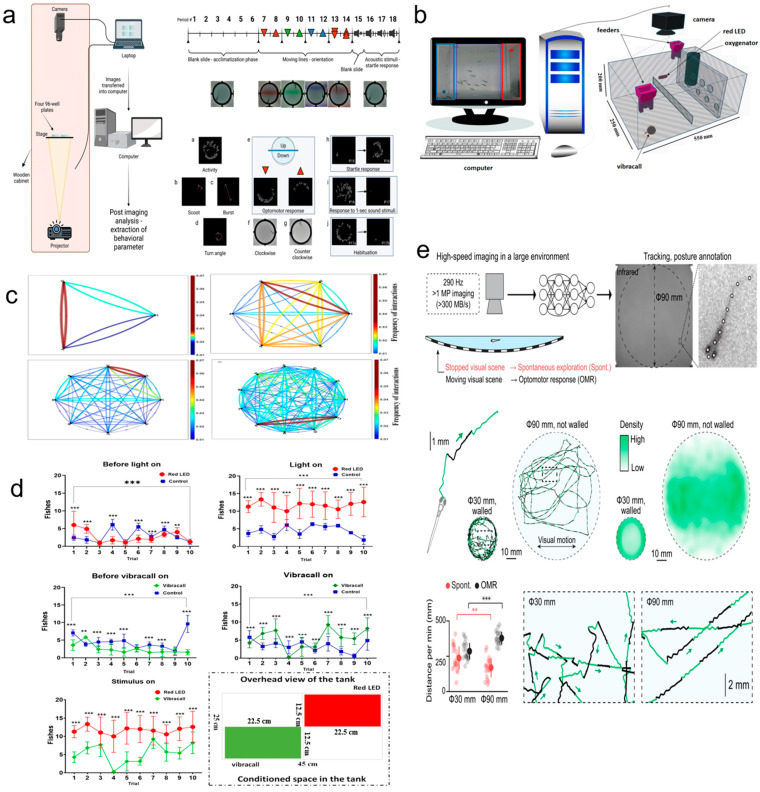
Techniques for behavioral research on zebrafish. (**a**) A high-throughput deep learning behavioral approach [165]. Open access. (**b**) Experimental configuration for measuring zebrafish learning [161]. Open access. (**c**) Zebrafish shoal interaction network. Mutual information was measured by the difference in the angle of the fish’s head during movement in the video [161]. Open access. (**d**) Zebrafish use a compound appetite-regulating response to two stimuli: red LED light and vibration [161]. Open access. (**e**) High-resolution and high-speed tracking of zebrafish behavior in large environment [158]. Open access. ** *p* < 0.001, *** *p* < 0.0001.

Technological advancements in video recording and analysis have enhanced behavioral assessments [158]. Three-dimensional behavior analysis is capable of analyzing more complex behavior characteristics and is also capable of finding the unique behavior characteristics of zebrafish and evaluating them [159]. Through the use of the chromatic fish analyzer, it is possible to detect small changes in behavior in real time from video images. Zebrafish behavior can be monitored by using this monitoring tool, and it is expected that zebrafish behavior can be analyzed more accurately; additionally, the impact on reproduction can be evaluated [160]. The research at present has proposed an automated system for evaluating zebrafish behavior in real time, which can adjust and evaluate zebrafish behavior in real time [161]. AI and machine learning, which have been rapidly developing, are increasingly being applied to zebrafish research. A deeper understanding of biological processes such as learning and memory can be gained through the analysis of zebrafish behavior [162]. In addition, automated systems for the real-time evaluation of zebrafish behavior are being developed, incorporating AI and machine learning to analyze high-throughput imaging data and understand the development of the nervous system (Figure 5) [163].

Furthermore, transgenic zebrafish models are increasingly used to manipulate and monitor the effects of stimuli on live zebrafish. It has been reported in previous studies that transgenic zebrafish can be used to manipulate and monitor the effects of olfactory stimulation on live zebrafish in order to assess how light impacts neuronal activity in the olfactory stimulation processing system of the transgenic fish. According to previous studies, olfactory activity plays an essential role in zebrafish reproduction. An understanding of the neurobiological mechanism of behavior change is helpful in understanding how external stimuli cause behavior changes [164]. In a similar manner, transgenic zebrafish are expected to be used for the assessment of reproductive toxicity based on behavioral measures. It is possible to establish disease models by introducing genes that are associated with specific reproductive behaviors, thus providing an invaluable platform for studying reproductive toxicity and understanding the neurobiological mechanisms underlying behavior changes.

## 6. Environmental and Public Health Implications

The increasing recognition of MNPs as a new form of pollution has significant environmental and public health implications. MNPs, found in water, soil, and the atmosphere, pose a potential threat to ecosystems and human health. Understanding their long-term impacts on aquatic organisms like zebrafish, as well as on terrestrial ecosystems and human health, is critical for the environment and public health. The pollution of aquatic ecosystems by MNPs may have widespread and profound effects, such as toxicity for aquatic organisms, propagation through the food chain, and the destabilization of aquatic ecosystems. These MNPs can also enter the human food chain, potentially causing chronic inflammation, immune system disorders, reproductive disorders, and an increased risk of cancer. There have been reports that MNPs are present in baby bottles and breast milk, where they may enter the infant’s body through blood circulation, where MNPs in humans may activate pathological features and cause reproductive toxicity [166,167,168]. Therefore, innovative approaches are necessary in order to better understand the effects of MNPs, including using advanced analytical techniques, biomarkers, and biological monitoring technologies. Developing biodegradable materials and environmentally friendly alternatives is also essential to mitigate these impacts.

Using zebrafish behavioral characteristics to assess the impacts of MNPs offers significant implications for public health and the environment. The rapid identification of behavior changes in zebrafish can aid research and monitoring efforts that require a quick assessment of MNPs’ impacts. Moreover, behavioral parameters provide a humane and sustainable means of evaluation, reflecting changes in the nervous and physiological systems and assisting researchers in understanding the mechanisms associated with MNPs. The study of MNPs’ effects on the reproductive system of zebrafish may provide insights into potential effects on human health. Given the similarities between zebrafish and human reproductive systems, examining the effects of MNPs on zebrafish germ cells, reproductive development, hormone levels, and behavior can infer potential impacts on human reproductive health. This necessitates the formulation of policies and measures to protect the environment and promote public health.

The results from zebrafish studies could contribute to establishing a risk assessment framework for MNPs, enhancing the protection of aquatic organisms and ecosystems. Determining the pathways, concentrations, and types of MNP exposure and assessing their effect on aquatic organisms and ecosystems are critical steps. The studies of zebrafish have revealed the mechanisms by which MNPs cause toxic effects on organisms, including bioaccumulation, endocrine disruption, and cytotoxicity in organisms. These insights inform environmental protection policies and suggest mitigation measures, such as improving wastewater treatment technologies, reducing sources of plastic pollution, and developing biodegradable materials.

In conclusion, the environmental and public health implications of our research on the reproductive toxicity of MNPs in zebrafish underscore the urgent need for comprehensive strategies to address the impact of MNPs. Implementing effective measures can reduce the entry of MNPs into aquatic ecosystems and protect both aquatic life and human health from their detrimental effects.

## 7. Challenges and Future Prospects

**Figure 6:**
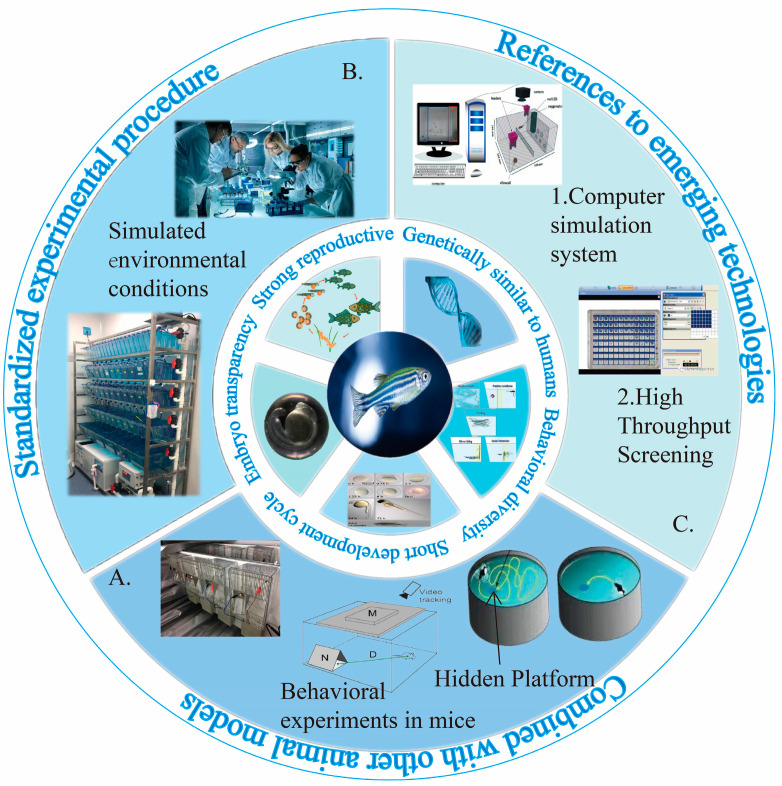
The advantages of zebrafish as model organisms and the improvement of behavior analysis techniques. (**A**) Combined with other animal models; (**B**) standardized experimental procedure; (**C**) references to emerging technologies.

Building on existing research results, it is essential to envision and identify areas that require further research and development to deepen our understanding and address the reproductive toxicity challenges posed by MNPs (Figure 6).

(a) Model enhancement and comparative research: Addressing the limitations of zebrafish as a nonmammalian model is essential. Future studies should focus on establishing validation mechanisms between zebrafish and other animal models, like mice or nematodes, and standardizing experimental procedures, like the Fish Embryo Toxicity test, to enhance reproducibility and comparability. This approach will provide a more comprehensive assessment of MNPs’ biotoxicity and their health effects.
(b) Bridging laboratory and Field studies: It is important to bridge the gap between laboratory studies and real-world conditions. Future research should incorporate a range of environmental factors, such as exposure time, concentration, and mixed toxicity with other pollutants, to better represent field conditions and understand the combined exposure effects of MNPs with other environmental pollutants.
(c) Advancing research with emerging technologies: There is a pressing need for rapid and accurate toxicity assessments, for example, with behavioral help, to support environmental policies and public health strategies. Future research should focus on long-term studies, multigenerational effects, and the early detection of biomarkers, especially finding and validating specific behavioral biomarkers. Integrating HTS, omics methods, and computational modeling will deepen our understanding of the mechanisms and risks related to MNPs. Global cooperation in research, the standardization of methods, and policy formulation is crucial to address the challenges posed by MNPs effectively.

## 8. Conclusions

This review systematically explored the complex reproductive effects and mechanisms of MNPs, highlighting the necessity for precise and sensitive assessment methods. Zebrafish, with their advantageous traits of their small size, rapid development, and ease of maintenance, have proven to be an ideal model for conducting behavioral tests to evaluate reproductive toxicity. Our analysis underscores the significant oxidative stress and hormonal changes caused by MNPs, especially their impact on the HPG axis and overall reproductive health. This work underscores the importance of behavioral analysis in zebrafish as a reliable method for assessing reproductive toxicity, offering a new perspective in understanding the broader implications of MNPs.

## Figures and Tables

**Table 1:** The effect of MNPs and their combination exposure with other contaminants on the reproductive system of zebrafish.

Classification	Coexposure	MP/NP Size	MNP Concentration	Exposure Time	Effects	Reference
PS-MPs	/	0.5 µm	500 µg/L	60 days	Ovarian tissue accumulation, ovarian cell apoptosis, decreased percentage of mature oocytes, changes in serum hormone levels (E_2_/T ratio), and changes in gene transcription along the HPG axis (*erα*, *cyp19b*, *fshβ*, and *lhβ*).	[84]
PS-MPs	/	1 μm	10, 100, or 1000 μg/L	21 days	Oxidative stress, gonadal histological changes (the thickness of the testicular basement membrane was significantly reduced), the level of apoptosis in testicular cells was significantly increased, and the expression of p53-mediated apoptotic pathways was detected.	[139]
PS-NPs	/	~70 nm	0.5 or 1.5 ppm	1 month	Gonadal bioaccumulation, disorders of lipid and energy metabolism, and oxidative stress.	[49]
PE MPs	/	10–22 μm, 45–53 μm 90–106 μm, 212–250 μm and 500–600 μm	2 mg/L	96 h	*Cyp1a* and *VTG1* expression levels were changed and the oogenesis process was interrupted.	[115]
NPs	Diethylstilbestrol (1, 10, and 100 ng/L)	70 nm	2 mg/L	21 days	GSI decreased and gonadal histological lesions inhibited the secretion of sex hormones (E_2_ and T) and VTG.	[100]
PS-NPs, PS-MPs	TPhP (0.5, 0.7, 1, 1.2, and 1.5 mg/L)	46 nm, 5.8 μm	2 mg/L	21 days	Exposure to MPs and NPs alone did not affect GSI in fish, while combined exposure of MNPS with TPhP exacerbated gonadal histological lesions and significantly increased VTG secretion.	[68]
PS-MPs	ZnO (1200 μg/L) ZnSO_4_ (500 μg/L)	5.0 µm	500 μg/L	30 days	Stronger oxidative stress, aggravated cell apoptosis. Improve antioxidant system and apoptotic responses.	[140]
PS-MPs	MC-LR (1, 5, and 25 μg/L)	1 μm	100 μg/L	100 μg/L	Gonadal histopathological changes, sex hormone changes (E_2_ and T), and HPG axis key genes’ (*gnrh2*, *gnrh3*, *fshβ*, *lhβ*, *cyp19a1b*, *cyp19a1a*, *erα*, *cyp11a*, *3βhsd*, *17βhsd*, *fshr*, and *lhr*) expression changes.	[129]
PA-MPs	Tris(1,3-dichloro-2-propyl) phosphate (TDCIPP) (0.4, 2, or 10 μg/L)	17.4 ± 7.2 μm	100 μg/L	4 months	Gonadal bioaccumulation.	[141]
PS-MPs	17α-Methyltestosterone (50 ng/L)	5 μm	0.5 mg/L	7, 14, and 21 days	The levels of E_2_, LH, and FSH were significantly decreased. Gonadal tissue damage, with significantly reduced expression of genes involved in gonadal hormone synthesis and metabolism *(cyp11a*, *cyp17a1*, *cyp19a1a*, *StAR*, *3β-HSD*, and *17β-HSD3*).	[101]

## Data Availability

Not applicable.

## Abbreviations

MPs	Microplastics
NPs	Nanoplastics
MNPs	Micro- and nanoplastics
ROS	Reactive oxygen species
PLA	Polylactic acid
HPG	Hypothalamic–pituitary–gonadal
NET	Norethindrone
PS	Polystyrene
TPhP	Triphenyl phosphate
VTG	Vitellogenin
T	Testosterone
PE	Polyethylene
EDCs	Endocrine-disrupting chemicals
AR	Androgen receptor
ERα	Estrogen receptor α
ERβ	Estrogen receptor β
E_2_	Estradiol
SOD	Superoxide dismutase
CAT	Catalase
GPX	Glutathione peroxidase
AChE	Acetylcholinesterase
LH	Luteinizing hormone
FSH	Follicle-stimulating hormone
HTS	High-throughput screening
